# Overweight in the Pacific: links between foreign dependence, global food trade, and obesity in the Federated States of Micronesia

**DOI:** 10.1186/1744-8603-2-10

**Published:** 2006-07-11

**Authors:** Susan Cassels

**Affiliations:** 1 Center for Studies in Demography and Ecology, University of Washington, Box 353412, Seattle WA 98195, USA

## Abstract

The Federated States of Micronesia (FSM) has received considerable attention for their alarming rates of overweight and obesity. On Kosrae, one of the four districts in the FSM, 88% of adults aged 20 or older are overweight (BMI > 25), 59% are obese (BMI > 30), and 24% are extremely obese (BMI > 35). Recent genetic studies in Kosrae have shown that obesity is a highly heritable trait, and more work is underway to identify obesity genes in humans. However, less attention has been given to potential social and developmental causes of obesity in the FSM. This paper outlines the long history of foreign rule and social change over the last 100 years, and suggests that a combination of dietary change influenced by foreigners, dependence on foreign aid, and the ease of global food trade contributed to poor diet and increased rates of obesity in Micronesia. The last section of the paper highlights the Pacific tuna trade as an example of how foreign dependence and global food trade exacerbates their obesity epidemic.

## 1. Background

Obesity and overnutrition are becoming major global health issues. In 2000, the World Health Organization stated that overeating is the "fastest form of malnutrition", and estimates that the number of people worldwide that are overweight and malnourished equals the number of people that are underweight and malnourished, at 1.1 billion people [1]. Body Mass Index (BMI) is the most common measure of body fat; BMI equals an individual's weight in kilograms divided by their height in meters squared. Nearly one in three Americans is obese (BMI > 30) and obesity rates have risen steadily over the last 40 years, from 13.3% to 30.5%. While such growth is concerning, these rates are not the highest in the world. On the island of Kosrae, a district in the Federated States of Micronesia (FSM), 88% of adults aged 20 or older are overweight (BMI > 25), 59% are obese (BMI > 30), and 24% are extremely obese (BMI > 35) [2].

Kosrae has received international attention for their alarming rates of obesity and has become the keystone study site for trying to identify genetic causes of obesity [2-5]. A census of the entire adult population of Kosrae has recently been completed, which included individual DNA samples, individual-level data on height, weight, blood pressure, and glucose levels, as well as information about the identity and medical status of family members. The goal of this ongoing work is to establish the possible relationship of genetic variation to human obesity. However, these studies note that Kosraean's have not always been overweight, and hint that changes in lifestyle and environment on Kosrae were coincident with increases in obesity. Much less attention has been given to these possible factors of obesity in the FSM. Most likely, changing social and environmental context along with unlucky genes are the main causes of Micronesia's obesity epidemic. The objective of this paper is to highlight potential contextual causes of obesity in Micronesia, specifically how a combination of dietary change influenced by foreigners, dependence on foreign aid, and the ease of global food trade contributed to poor diet and increased rates of obesity in Micronesia.

**Figure 1:**
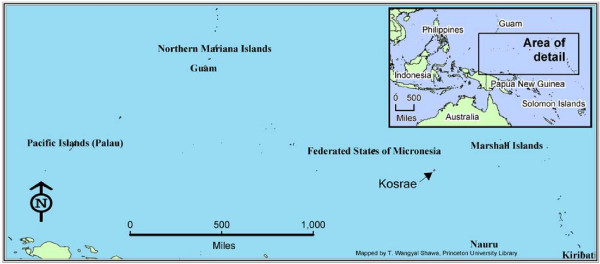
Map of the Federated States of Micronesia.

Micronesia, a country comprising of more than 600 islands in the Central Western Pacific, has a long history of foreign influence and dependence. (See Figure 1 for a map of Micronesia). Spain was the first nation to colonize Micronesia; they arrived in 1886 and controlled the islands until Germany took over in 1899. The Japanese arrived 15 years later and built a thriving economy in Micronesia up until WWII. In 1945 the United States occupied the islands and soon became the "administering authority" of the U.N. Trust Territories of the Pacific Islands (i.e. Micronesia). The Federated States of Micronesia (FSM) did not become an independent nation until 1986. However, they continued to receive considerable aid from the U.S. through an agreement called the Compact of Free Association. Between 1986 and 2003, the FSM received US$1.5 billion in aid from the U.S. The Compact was renewed in 2004, and the FSM has been promised US$2.1 billion in aid and assistance over the next 20 years.

Micronesia was isolated for a long time, but then experienced significant changes in the last hundred years. Especially in the last fifty years, the population has been significantly influenced by the U.S., particularly in regards to diet. They have been and still are extraordinarily dependent on foreign nations for development and imported food. And finally, they are one of the most – if not *the *most – overweight populations in the world. Thus, Micronesia is an interesting place to study the links between foreign dependence, global food trade, dietary change, and obesity.

Worldwide, developing nations have experienced dietary change associated with modernization and development. The next section is a review of these links. Following that section, the focus returns to the Pacific to identify the associations between dietary change, foreign influence and trade, and obesity in Micronesia, especially over the last fifty years. The last section of the paper details the Pacific tuna trade to highlight these links between global food trade, foreign dependence, diet and obesity in Micronesia. The state of the Pacific tuna industry contributes to Micronesia's increased reliance on imported food, unhealthy diet, and population health problems.

## 2. Modernization and dietary change in developing countries

Many have studied the role of modernization in dietary change and obesity in the developing world [6-10]. These studies have suggested that rapid changes in diets resulting from modernization (i.e. improved standards of living and continued development) and market globalization have had a significant impact on the nutritional status of populations. For instance, some work has shown that modernization is associated with increased consumption of salted and processed foods and animal foods higher in saturated fat, and decreased consumption of complex carbohydrates [8,11,12]. Increased reliance on imported foods rather than traditional foods is also associated with modernization [7,13]. With the ease of global food trade, food preference may not be sufficient to ensure a healthy diet. The low cost and wide availability of imported foods, especially high-fat meat products, result in nutritionally detrimental decisions to consume cheaper, nutrient-poor foods rather than healthier alternatives, such as fish.

This is a relatively new development. Thirty years ago, lower incomes were associated with lower fat, lower animal protein, and higher complex carbohydrate intakes; when incomes increased, so did consumption of total and animal fat. However, this traditional correlation between income and diet has changed recently with globalization of food production and trade [14]. The global value of food trade grew from US$224 billion in 1972 to US$438 billion in 1998; food now accounts for 11% of global trade [15]. Along with the global food trade, people's preferences and increased food availability has influenced dietary patterns. Due to the widespread availability of low cost fat, even people from lower income countries consume a higher percentage of calories from fat.

Many countries, especially in the Pacific, have become dependent on trade in the global market. While global trade has brought some improvements in the standard of living and access to health care and services, for example, but has also induced many negative consequences. A significant negative consequence of global trade for countries in transition is an inappropriate, unhealthy diet high in saturated fat and low in complex carbohydrates [16], and a rise in obesity rates.

The connection between modernization, market globalization and obesity has been empirically documented. A study comparing Pima Indians living in rural Mexico (a traditional lifestyle) with genetically identical Pima Indians living near Phoenix in the U.S. (a more modernized lifestyle) showed that the American Pimas had an average BMI 10 points higher than their rural Mexican counterparts [6]. Another interesting comparative study looked at American and Western Samoans to explore the differences in dietary intake and health consequences [11]. Mean BMI for American Samoans in their study, who live a more modern lifestyle, was 35.2, compared to 30.3 for Western Samoans. Lastly, a study in Papua New Guinea demonstrated that more modern Papuans had higher mean BMI and lower levels of physical activity [8]. These studies suggest that aspects of modernity are associated with physical inactivity and increased availability of energy-dense Western food, which increases the risk of obesity.

A comparative study of Micronesians living in traditional and modern settings in 1970 also illustrates the association between modernization and dietary change [17]. The study showed the dietary difference between Micronesians living a traditional way of life in Palau and Micronesians living in a more modern economy in Guam and California, which essentially foreshadowed dietary change that accompanied modernization in the FSM since 1970. Total energy intake was greater for the traditional-lifestyle groups, but the proportion of energy coming from fat – mostly saturated fat – was much higher in the modern-lifestyle groups. The traditional-lifestyle groups relied more on energy from carbohydrates than the modern-lifestyle groups, with the predominant carbohydrate sources as follows: taro and cassava in Palau, rice and bread in Guam, and bread in California. Total protein intake did not vary drastically between the two groups, but the type of protein was different. The modern-lifestyle groups relied mostly on meat and poultry, and the traditional-lifestyle groups consumed more fish. Diets in the FSM today resemble the diets of Micronesians living in Guam and the U.S. in 1970: more simple carbohydrates, saturated fat, and imported meat.

**Figure 2:**
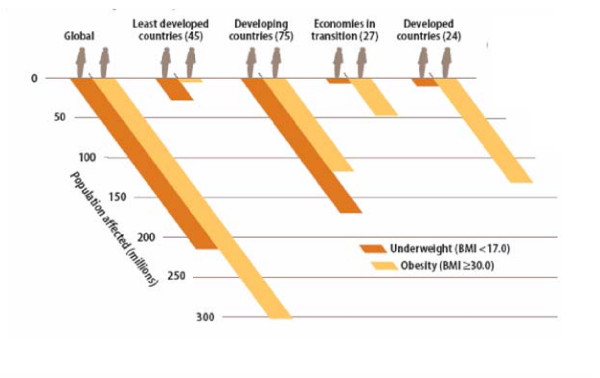
Adult population affected by underweight and obesity by level of development (estimates for the year 2000).

The nutritional transition – the shift toward refined foods, meat and dairy products with high levels of saturated fats – along with reduced energy expenditure has contributed to the global rise in obesity. This has led to a shift in the global burden of disease. It is estimated that within five years, two-thirds of the global burden of disease will be attributable to non-communicable disease associated with diet [15]. This change can be seen in the ratio of underweight to obese populations in economies in transition (see Figure 2) [18]. In least developed and developing countries, underweight and malnourished populations surpass obese populations, but in economies in transition and developed countries, obese and malnourished populations pose more of a threat. Obesity is truly becoming a global disease burden, and one does not need to look much farther than Micronesia to see evidence.

## 3. Foreign influence and dietary change in Micronesia

In the late 1800's after sustained Western contact with explorers, traders, and missionaries, Micronesia saw almost a century of colonial rule, starting with Spain in the late 1800's and ending with the U.S. in the 1980's, that influenced local diets. Today, the U.S. and FSM still have an agreement called the Compact of Free Association. Under this agreement, the U.S. provides economic assistance and other federal benefits to the FSM, and in turn can use the Micronesian islands for defense and military operations. One externality of this agreement is continued dependence on the U.S. for aid and subsidies.

Dietary preference and food availability has changed since pre-Western contact. Traditional and local diets comprised of plant foods such as taro, breadfruit, yams, coconut, arrowroot and bananas, and animal foods were mostly freshwater, reef, and pelagic fish, crustaceans, and possibly fruitbat and birds. The Spaniards introduced maize, cassava, sweet potatoes, chickens and pigs [19], and rice became a staple after the Japanese occupation. Thus, traditional diets changed slightly with Spanish, German and Japanese influence, but there was little evidence of malnutrition until the American occupation [20].

Food consumption changed drastically in the late 1960's and 1970's, which was closely tied to the start of U.S. subsidies [21]. There was very slow growth and limited U.S. activity with or within Micronesia during the first 20 years after the war. U.S. subsidies to Micronesia started in 1962 at US$6 million a year, and increased quickly to US$130 million in 1978. With the increase of subsidies from the U.S. came salaried employment in the FSM; the per capita income rose from US$60 to US$400 in the same time frame. Concurrently, the proportion of global food production and trade increased enormously. Therefore, the new cash-based economy in Micronesia triggered a significant shift in lifestyle; one major difference was imported foods became more accessible and affordable [21].

Dietary studies since WWII in the FSM illustrate the change in diet over the last fifty years. In the 1950's, there was a strong reliance on local foods [22]. By the 1970's, less local foods were consumed and the main energy sources were from rice and imported foods. Fish was still eaten often, but "empty calorie" imported foods were becoming more common [23,24]. The United States Department of Agriculture (USDA) supplementary feeding program, which started in the 1960's, increased in the 1970's, and continued through the early 1990's, significantly influenced Micronesian's eating habits as well. This program provided school lunches mostly consisting of rice and tinned foods. In 1985, the school lunch program provided meals for 30% of the population every other day of the year. Many suggest that this program increased food dependency on the U.S., shifted food tastes, and contributed to local, healthy foods being replaced with rice, refined carbohydrates, and tinned foods [20]. Consumption of sugar and sweet foods also increased in the 1980's [25]. Most recently, nutritional studies have found that local and canned fish, imported chicken and turkey tails are the major protein foods [26,27].

Today, rice, wheat flour, sugar, refined foods, and fatty meats such as corned beef, turkey tails, and spam are commonly eaten in the FSM due to many interrelated factors such as convenience, affordability, taste and prestige. First, the FSM has suffered a great loss of food production because of inconsistent external and internal government policies and unplanned externalities from U.S. food aid programs [20]. The tuna industry, which will be described in more detail in the following section, is a telling example of how inconsistent government policies influenced local food production. Second, an overwhelming onslaught of imported foods has reached Micronesia starting in the 1960's. In 1986 food and beverages imports accounted for 40% of the total value of imports to Micronesia; these imported foods were not essential or without local substitutes, and many of the food products were nutritionally harmful [20]. Lastly, throughout Micronesia there has been an erroneous belief that imported foods were superior to local foods. American influence has changed both the preference and availability of foods over the last half of the 20^th ^century.

Turkey tails are a telling example of inferior imported foods replacing healthy local foods. According to the Food and Agricultural Organization (FAO), consumption of poultry meat in the Pacific has increased from an average of 19 kg per capita per year in 1980 to 34.4 kg per capita in 2002 [28]. In the U.S., the tails of turkeys are deemed inedible, but exporters found a market for them in Micronesia. Frozen imported turkey tails – simply gristle and fat – cost under $1 a pound, are commonly eaten in Micronesia, and are extremely unhealthy.

## 4. Obesity prevalence in Micronesia over time

Obesity is a fairly new phenomenon in Micronesia. Historically, Pacific Islanders were not overweight, as illustrated by documents from early explorers' observations. Chronicles of Magellan (1521) and Quiros (1606) refer to Pacific Islanders as "singularly tall, muscular and well-proportioned people" [29]. French explorer Louis de Bougainville said "I never saw men better made" after visiting Tahiti, and Captain James Cook (1770's) described many of the Pacific island populations as having good diets and health [30].

In Micronesia, diets may have been influenced by foreign rule since the Spanish occupation, but signs of overweight or obesity were not evident before the U.S. occupation. Documentation and photographs from the German South Seas Expedition (1909–1910) show the Micronesians as lean and healthy [31]. Additionally, the U.S. navy conducted a health survey in Micronesia after WWII (late 1940's) and noted almost a complete absence of obesity, hypertension, or diabetes [21].

The rise in obesity began at the same time as the U.S. subsidies reached Micronesia in the 1960's and 1970's. As previously mentioned, there was a complete shift in the local lifestyle with the new cash-based economy. Micronesians no longer needed to collect firewood because they could use their new propane stoves for cooking. They no longer needed to work the land for food, because with money, food could be bought at the store. These lifestyle changes, besides contributing to dietary change, also made exercise unnecessary [21]. Thus, the combination of a poor diet and less exercise resulted in a rise in obesity rates.

**Figure 3:**
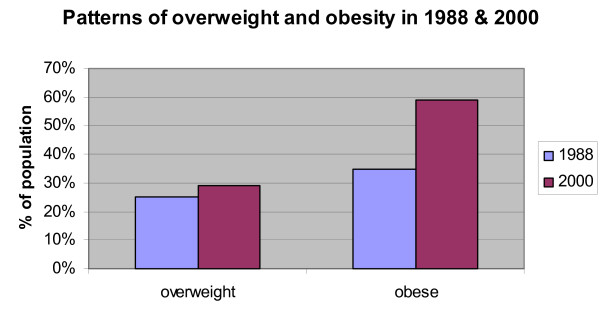
Overweight and obesity prevalence in Kosrae, FSM in 1988 and 2000.

The first complete study of overweight and obesity in FSM was the National Nutritional Survey of the Federated States of Micronesia in 1987/1988 [25]. Comparing their results with a study of obesity conducted in 2000 on Kosrae [2], one of four states in the FSM, shows a substantial increase in overweight and obesity prevalence (Figure 3). Prevalence of overweight (BMI 25 – 30) increased from 25% in 1988 to 29% in 2000. The increase in obesity was even more drastic. In 1988, 35% of adult Kosraeans were obese (BMI > 30) compared to 59% in 2000. Two caveats must be noted when comparing these studies. First, the 1988 study only reports overweight and obesity prevalence for women aged 15 – 49. However, the results from the 2000 study are for men and women aged 20 – 85; the average age was 42. Average female BMI was 31.7 (+/- 5.9) in the 2000 study, slightly higher than the average male BMI at 30.1 (+/- 5.2). Therefore, the comparison over time should be viewed with caution: excluding men might overestimate overall obesity prevalence (1988 study) since women have a higher average BMI, but since obesity increases with age, the 2000 study might also overestimate obesity prevalence – compared to 15 – 49 year olds – because it uses an older sample.

A clear rise in overweight and obesity in Pacific Island populations occurred in the second half of the 20^th ^century [32], which has been attributed to economic modernization and associated dietary change. The diet on Kosrae and the other states in the FSM became more Westernized during the U.S. occupation. High-fat foods imported mostly from the U.S. were being consumed in large quantities, and these dietary changes led to dramatically increased prevalence of overweight and obesity.

The following section examines the role of the tuna industry in Micronesian behavior and health. The current state of the Pacific tuna trade highlights the role of global food trade and foreign dependence in changing food production, consumption, and obesity in Micronesia.

## 5. The role of the tuna industry in the Micronesian obesity epidemic

Tuna fishing in the Central Western Pacific is a US$2 billion dollar a year industry. However, the FSM, which is located in the middle of these rich tuna stocks, has never been able to compete globally in the industry. Instead, Micronesia sells their fishing rights to foreign nations for a fraction of its worth. These other nations then trade tuna globally, some of which eventually returns to Micronesia. This story highlights how the FSM continues to be dependent on foreign nations due to insufficient internal development; this leads to further dependence on foreign nations for food imports.

Fish and marine resources have traditionally been an important component of the Micronesian diet. Fresh fish – usually reef fish – is still eaten when available; otherwise, it is substituted with canned fish [27]. Despite the abundance of fish off shore, fresh fish is consumed less today than it used to be [33]. Data from a 1997 household income and expenditure survey estimate that fish consumption ranges from 72 to 114 kilograms per person per year, and canned fish comprises about 25% of this consumption [33]. Imports of canned pelagic fish (mostly tuna and mackerel) have increased drastically over the last 10 years. According to the Food and Agricultural Organization (FAO), the FSM imported 242 metric tons of canned fish in 1992; by 2001 the figure increased to 1,369 metric tones [28]. Thus, much of the fish that Micronesian's eat has been processed elsewhere and imported into the country.

**Figure 4:**
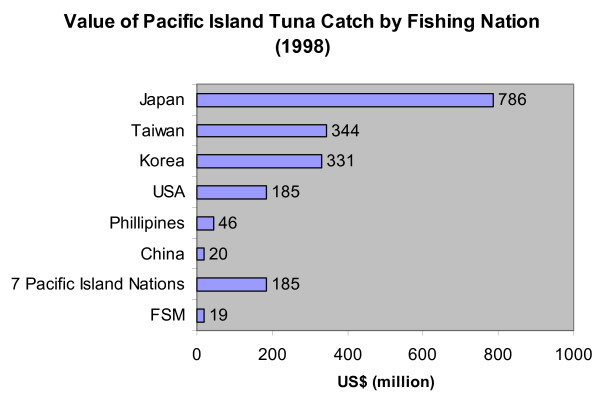
Value of Pacific Island tuna catch by fishing nation, 1998.

The FSM has jurisdiction over the fishing areas off of their shores, which includes some of the richest tuna stocks in the world. This jurisdiction came from the 1977 Law of the Sea Convention, which created a 200-mile Exclusive Economic Zone (EEZ) off of nations' shores. The FSM declared their EEZ in 1979, which covers almost 3 million square kilometers in the Central Western Pacific. The tuna stocks in the Central Western Pacific (the ocean immediately surrounding the FSM) have an estimated value of US$2 billion [34]. The vast fishery supplies about half of the world's canned tuna market, and about one third of the total tuna supply. Many foreign vessels exploit the tuna stock in this area. Japan – for long the world's largest harvester as well as consumer of tuna – harvests more than 90% of its tuna in the Pacific, and nearly 40% from the Central Western Pacific in 1995 [35]. Around 68% (US$1.3 billion) of the total tuna catch in the Central Western Pacific was taken from within Pacific Island Countries' Exclusive Economic Zones; the Pacific Island Countries include the Solomon Islands, Papua New Guinea, Vanuatu, Samoa, Fiji, Kiribati, and Tonga. However, actual Pacific Island Nations only harvested around 11% of the total catch (Figure 4) [34]. The FSM does not have sufficient infrastructure for a globally competitive fishing industry. Instead of fishing the tuna stocks themselves, the FSM sells their fishing rights to foreign nations.

The FSM commenced fishing access agreements with Japan in 1981, and began to sell their fishing rights. As previously mentioned, Japan had large stakes in the Pacific tuna industry at that time. Initially, access fees followed a lump-sum system, but soon switched to a per-vessel per-trip system. The per-vessel system was more accurate and fees could be based on actual catch. In the early 1980's, the rate of return from the access fees was set between 3 – 4% of the catch value, but in reality it was significantly lower. The low access fees were attributed to a number of factors: 1) lack of any real scarcity value – access agreements did not set a limit to the catch; 2) the small number of buyers; 3) the relatively large number of sellers; and 4) the inability to enforce compliance with agreements or monitor the value of the catch. Thus, the amount of money that the FSM receives from fishing-rights fees is much less than the potential value of the tuna, given that the FSM could harvest and sell the tuna in the global market.

**Figure 5:**
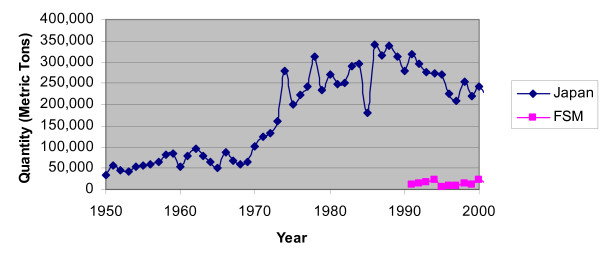
Total fishery (tunas, mackerel, billfish) production in the Central Western Pacific.

**Figure 6:**
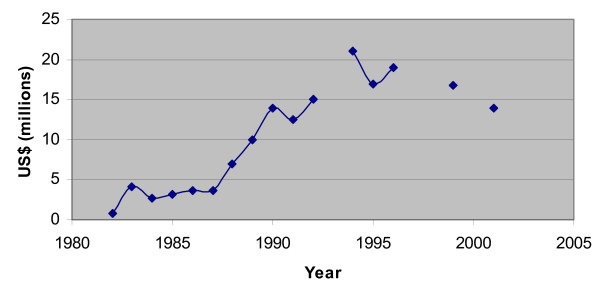
Amount of fees received by FSM for fishing rights in its EEZ.

Depicted in Figure 5, the FSM did not harvest much tuna compared to Japan [28]. In 1998, the FSM harvested only 1% of the total tuna catch in the Central Western Pacific. However, the FSM collected about $170 million in fees for its tuna fishing rights from 1979 to 2000. The fees contribute to anywhere between 20 – 30% of the total domestic revenue in a given year, a significant portion [36]. Figure 6 shows the annual fees collected by the FSM for the last twenty years [37-39]. Note that some years are missing; the fishery access agreements and fees between individual nations and fishers are difficult to attain. In the mid 1990's, foreign commercial fishing fleets paid over US$20 million annually for the right to operate in FSM territorial waters, with Japan the largest customer. However, this figure has recently dropped to about US$13 million. In 1998, 75% of the fees were paid by Japan.

As previously mentioned, the value of the Central Western Pacific tuna industry is near US$2 billion. Pacific Island nations, as a whole, only receive around US$70 million in fees, or 3.5% of the total value. Thus, they receive an extraordinarily small amount of income relative to the value of the industry. Micronesians do not benefit with employment either; only about 150 FSM citizens work on foreign tuna vessels at any given time [33]. Most importantly, they do not benefit by receiving fresh, local tuna.

In FSM's EEZ in 1999, 130,000 tons of tuna was harvested (down from 230,000 in 1995), and only 2% of the catch was from local Micronesian vessels. The majority of fish landed by the small locally-based longline vessels is exported to Japan via Guam. Fish exports account for more than 90% of total exports. Fish that are not export-quality, about 20%, are sold locally to processors who produce value-added products for export, or to restaurants [33]. The amount of fish that enters the domestic food supply translates to about 0.25% of the total tuna catch in the Central Western Pacific [34].

Subsistence fishers are still active in the FSM, mostly exploiting inshore resources and selling excess catches through various local outlets. However, attempts to develop and structure small scale fisheries have met limited success [40]. To date, no viable fishery operating in the FSM has reached its full potential despite more than US$70 million in investments [30,41]. For example, in 1995 US$6.5 million was loaned to the FSM from the Asian Development Bank for developing a fleet of locally-owned longline vessels targeting the fresh sashimi market. In 2001, the Micronesian Longline Fishing Company was founded, but has never been profitable.

Micronesians are essentially selling their own natural food resources for a fraction of the true value, and then using the revenue to import nutrient-poor food from the U.S. The FSM does not have the infrastructure to realistically compete in the global tuna market. Thus, the current structure of the Pacific tuna industry is an example of how lack of development (partly due to the U.S subsidies and U.S. dependence) has lead the FSM to continue to be dependent on foreign nations. The cash-economy stemming from the tuna industry contributes to the continued cycle of food dependence, imported-food, and poor diet, which is partly responsible for Micronesia's unhealthy, obese population.

## 6. Conclusion

As an economy still in transition, Micronesian's reliance on a cash-economy but lack of self-sufficiency puts them in a precarious position to depend on imported food. A typical grocery store in Micronesia today is stocked with imported nutrient-poor, canned and packaged foods. White bread, sugar, canned goods and processed foods, and canned and frozen meats such as spam, corned beef, hot dogs, and turkey tails dominate the shelves. It is estimated that the average household spends 38% of its income on imported foods [30]. Even at traditional weddings, funerals, and other cultural events, imported foods are found. Fresh fish, bananas, and coconuts used to be essential in these exchanges, now store-bought food is brought as gifts [42]. This current lifestyle is due to a long history of foreign influence and dependence, along with enhanced global food trade, and has confounded any unlucky genetic vulnerabilities of obesity in the FSM.

However, with the spotlight on Micronesia's obesity epidemic partly due to the genetic research taking place on Kosrae, Micronesian's attitudes toward obesity are slowly changing. Many health professionals in the Pacific Islands are now emphasizing eating traditional foods and encouraging residents to get back to a healthy lifestyle and to their cultural roots. With the US$2.1 billion in aid from the Compact of Free Association gone in twenty years, the FSM will have no choice but to invest in some internal development, promote self-sufficiency, and incite significant lifestyle changes.

## Competing interests

The author(s) declare that they have no competing interests.
